# Exploring the acceptability of a brief online theory-based intervention to prevent and reduce self-harm: a theoretically framed qualitative study

**DOI:** 10.1192/bjo.2022.568

**Published:** 2022-10-12

**Authors:** Chris Keyworth, Leah Quinlivan, Jessica Z. Leather, Christopher J. Armitage

**Affiliations:** School of Psychology, University of Leeds, UK; NIHR Greater Manchester Patient Safety Translational Research Centre, University of Manchester, UK; NIHR Greater Manchester Patient Safety Translational Research Centre, University of Manchester, UK; and Manchester Centre for Health Psychology, University of Manchester, UK; NIHR Greater Manchester Patient Safety Translational Research Centre, University of Manchester, UK; Manchester Centre for Health Psychology, University of Manchester, UK; and Manchester Academic Health Science Centre, Manchester University Foundation Trust, UK

**Keywords:** Self-harm, qualitative research, suicide, patients, primary care

## Abstract

**Background:**

The volitional help sheet for self-harm equips people with the means of responding automatically to triggers for self-harm with coping strategies. Improving acceptability may be crucial to increasing effectiveness and reach. The Theoretical Framework of Acceptability (TFA) was developed to guide the assessment of intervention acceptability, but to date, no studies have applied the TFA to understand acceptability of interventions for self-harm.

**Aims:**

To apply the TFA to (a) explore people's experiences of a brief intervention to reduce repeat self-harm; and (b) understand the most prominent aspects of intervention acceptability, to make recommendations for intervention refinements and successful implementation.

**Method:**

Sixteen semi-structured interviews were conducted with people who had previously self-harmed. The TFA informed a framework analysis in which findings were mapped onto the TFA.

**Results:**

Four TFA domains were identified that were associated with acceptability of the volitional help sheet for self-harm: affective attitude, burden, intervention coherence and perceived effectiveness. People were generally positive about using the volitional help sheet (affective attitude), understood the volitional help sheet and how it worked (intervention coherence), highlighted engagement as a motivating factor in using the volitional help sheet (perceived burden) and described how the volitional help sheet could be implemented by healthcare professionals (perceived effectiveness).

**Conclusions:**

Further modifications could still be made, but it is hoped that this intervention provides a useful tool for individuals to construct their own personalised implementation intentions, and as part of longer-term support for preventing self-harm as delivered by healthcare professionals.

Clinical care for people who have harmed themselves is an important issue. Self-harm includes ‘any act of self-poisoning or self-injury carried out by an individual irrespective of motivation’,^1^ and is associated with suicide; therefore, developing preventative strategies is vital.^2,3^ Reasons why people engage in self-harm may include triggers such as feelings of defeat or entrapment^4^ that increase the urge to self-harm. Providing people with a means of responding to such critical situations by forming automatic coping plans may lessen the likelihood of self-harm occurring.

A brief intervention for self-harm, based on implementation intentions,^5^ may help people to automatise coping responses to triggering critical situations. Implementation intentions are ‘if-then’ plans, which draw people's attention to critical situations (‘if’) and provides them with an appropriate response to those situations (‘then’) that spring to mind automatically when ‘if’ is encountered. This ‘volitional help sheet’ has been shown to be effective in reducing self-harm in people recently admitted to hospital for self-harm.^6^ However, study findings were limited by high rates of attrition at follow-up and a lack of acceptability testing. Consequently, it is necessary to explore acceptability further, to improve its effectiveness for reducing self-harm.

Intervention acceptability is an important consideration of complex intervention development,^7,8^ with successful implementation and intervention effectiveness likely to be dependent upon perceptions of acceptability.^8,9^ For example, interventions perceived as acceptable by those delivering and/or receiving them are more likely to result in favourable outcomes, including treatment adherence,^10^ support for public health policy^9^ and acceptance of behaviour change interventions.^11^ However, exploration of intervention acceptability has been limited by an *ad hoc* approach and a lack of standardised tools to ensure that all aspects of acceptability can be fully explored.

The Theoretical Framework of Acceptability (TFA)^8^ is helpful in this regard, and was developed to guide the assessment of the acceptability of interventions as a multifaceted construct.^8^ The TFA comprises seven domains: affective attitude (how individuals feel about taking part in an intervention), burden (the amount of effort required to engage with an intervention), perceived effectiveness (whether individuals perceive an intervention as likely to achieve its purpose), ethicality (the extent to which an intervention fits with individuals’ personal values), intervention coherence (whether individuals understand an intervention and how it works), opportunity costs (what is given up, such as time, to take part in an intervention) and self-efficacy (how confident individuals are doing the intervention). The advantage of using the TFA, as opposed to more general approaches to investigating acceptability, is that the TFA allows a more comprehensive, rich and varied assessment of intervention acceptability, and allows opportunities to make iterations to interventions based on specific domains of the TFA.^12^

Aims of the present study

The TFA has previously been used to guide data collection and data analysis in qualitative studies exploring acceptability with respect to a range of long-term health conditions^13–15^ and health behaviours.^16^ Studies have explored acceptability of interventions for self-harm generally, including acceptability of text-based interventions to support adolescents at elevated suicide risk,^17^ and a problem-solving training intervention for self-harm in prison settings.^18^ However, to date, no studies have applied the TFA to understanding acceptability of interventions for self-harm. Consequently, we aimed to apply the TFA to (a) explore people's experiences of a brief intervention to reduce and prevent repeat self-harm; and (b) understand the most prominent aspects of intervention acceptability, to make recommendations for intervention refinements and successful implementation.

## Method

### Design and participants

We conducted a qualitative study, using semi-structured telephone interviews. Participants had previously taken part in a large cross-sectional survey examining the acceptability of a brief behaviour change intervention to help support people to reduce repeat self-harm (Clinicaltrials.gov identifier: NCT04420546).^19^ All participants had a history of self-harm.

### Procedure

**Fig. 1:**
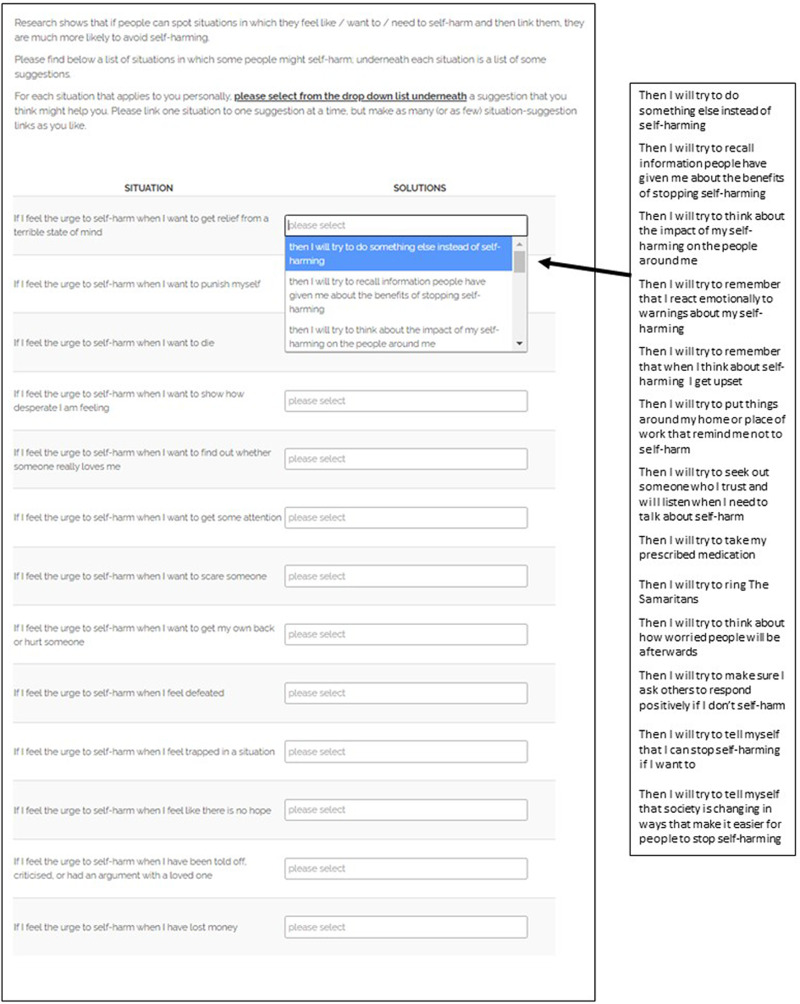
The volitional help sheet for self-harm.

The authors assert that all procedures contributing to this work comply with the ethical standards of the relevant national and institutional committees on human experimentation and with the Helsinki Declaration of 1975, as revised in 2008. All procedures involving human patients were approved by the University of Manchester Research Ethics Committee (reference number 2020-8446-15312), and informed consent was obtained from participants. The volitional help sheet provides people with a list of critical situations where the urge to self-harm may be heightened, and a list of coping responses designed to decrease the likelihood of self-harming.^6,19,20^ Briefly, the volitional help sheet provides a theoretically driven framework for participants to construct their own implementation intentions, drawing on theories of suicidal behaviour,^21^ self-harm motivation literature^22^ and the transtheoretical model of change.^23^ Participants formed implementation intentions by linking critical situations (‘If I feel the urge to self-harm when I feel trapped in a situation … ’) with appropriate responses response (‘ … then I will try to make sure I ask others to respond positively if I don't self-harm’) by choosing an appropriate response from a drop-down menu for each critical situation. Participants were free to make as many situation–response links as they desired. A screenshot of the volitional help sheet is provided in Figure 1, for illustrative purposes.

Initially, advertisements were placed on forums for self-harm, inviting people to take part in an online survey. The volitional help sheet for self-harm, developed into an online format, appeared at the end of the questionnaire. After taking part in the survey, participants were invited to take part in the qualitative study. As one of the main study aims was to make recommendations for intervention refinements and successful implementation, all participants were eligible to take part in the interview, regardless of whether they successfully formed an implementation intention as part of the intervention. We selected a convenience sample, whereby participants who agreed to take part provided their contact details so that a member of the research team could arrange a suitable time to conduct the interview. Participants provided written informed consent before the interviews, which were audio-recorded and transcribed verbatim. Interviews were conducted by one of the study authors (C.K., who is trained in conducting qualitative interviews), using a topic guide (presented in Supplementary File 1 available at https://doi.org/10.1192/bjo.2022.568) that probed participants’ experiences of using the volitional help sheet for self-harm. A topic was developed (see Supplementary File 1) to probe each of the seven constructs of the TFA (described above): affective attitude, burden, perceived effectiveness, ethicality, intervention coherence, opportunity costs and self-efficacy. Data collection ceased when the research team agreed by consensus that no new themes were emerging from the data.

### Analyses

A directed content analysis approach, which is suitable when the research uses an existing theoretical framework to interpret the data, was used to identify and categorise instances of the TFA domains.^24,25^ Principles of the framework approach^26^ were used to inform data analysis. First, deductive coding was used to organise the data in line with each of the TFA domains. Directed content analysis was used to identify and categorise instances of TFA domains.^27^ This involved reading each transcript and coding occurrences relating to each TFA domain.^28^ Analysis involved coding each occurrence in the interviews of each of the seven TFA domains, using the definitions accompanying each domain.^8^ This was done for all TFA domains. Second, inductive coding comprised generating explanatory themes in line with the most prominent TFA domains identified from the first level (deductive) coding. Specific codes within each TFA domain were grouped into themes. Initial codes were generated and collated into potential themes by C.K., who shared the coding framework and key illustrative quotes with C.J.A. as the analysis progressed. Any areas of contention were discussed and themes were refined accordingly, to ensure trustworthiness of the data. All authors were involved in finalising the main themes. An NVivo file (version 12 for Windows, QSR International, Burlington, MA, USA, https://www.qsrinternational.com/nvivo-qualitative-data-analysis-software/support-services/nvivo-downloads) was used to organise the data. The codes focused on different aspects of acceptability with respect to using the volitional help sheet to reduce repeat self-harm, according to each TFA domain.

## Results

### Sample characteristics

Participant demographics are presented in Table 1 (reported by participants as part of the cross-sectional survey). Social grades are presented according to the National Readership Survey classification system, according to occupation: higher managerial and professional (A); intermediate managerial and professional (B); supervisory and professional (C1); skilled manual workers (C2); semi-skilled and unskilled manual workers (D); and casual or lowest grade workers, pensioners and others who depend on the welfare state for their income (E).

**Table 1:** Sample characteristics

Participant number	Gender	Age (years)	Ethnicity	Social grade	Suicidal ideation	Suicidal attempt	Non-suicidal self-harm
p01	Female	29	White British	C2	The past week	Over a year ago	The past year
p03	Female	39	White British	E	The past year	The past year	The past year
p04	Female	48	White British	C1	The past week	The past year	The past week
p08	Non-binary (she/they)	23	White British	D	The past week	The past year	The past week
p09	Female	21	Not reported	Not reported	Not reported	Not reported	Not reported
p10	Male	49	White British	Not reported	The past year	The past year	The past year
p11	Female	41	White British	C2	The past year	Over a year ago	Over a year ago
p12	Male	59	White British	C1	The past week	The past year	The past week
p13	Female	27	White British	C1	The past week	Over a year ago	Over a year ago
p14	Male	45	Any other Asian or Asian British background	A	Over a year ago	Over a year ago	The past year
P16	Female	33	White British	B	Not reported	Not reported	The past week
p18	Transgender male	26	White British	D	Over a year ago	Not reported	The past week
p19	Male	59	White British	C1	The past week	The past year	The past week
p20	Female	26	White British	E	The past year	Over a year ago	The past year
p21	Female	28	White British	Not reported	The past year	The past year	The past year
P23	Non-binary	27	White British	E	The past week	Over a year ago	The past week

Social grades were assigned according to the National Readership Survey classification system, whereby the general population are assigned social codes according to occupation: higher managerial and professional (A); intermediate managerial and professional (B); supervisory and professional (C1); skilled manual workers (C2); semi-skilled and unskilled manual workers (D); and casual or lowest grade workers, pensioners and others who depend on the welfare state for their income (E). History of self-harm is reported as either self-harm in the past week or self-harm in the past year, not both. Self-harm in the past year relates to all other weeks in the remainder of the year (weeks 2–52).

The final sample (*n* = 16), recruited from a total of 51 people who were happy to be contacted to take part in the semi-structured interview. When asked their gender, nine persons stated they were women, four stated they were men and three stated their gender as ‘other’, with variations in age (range 21–59 years, mean age 36 years) and social grade (A: *n* = 1; B: *n* = 1; C1: *n* = 4; C2: *n* = 2; D: *n* = 2; E: *n* = 3; not reported: *n* = 3). Participants’ ethnic backgrounds were predominantly White British (*n* = 13). Thirteen of the sixteen (81.3%) participants formed at least one implementation intention and therefore engaged with the intervention.

With respect to history of self-harm (presented in Table 1), seven participants (43.8%) reported a suicidal attempt in the past year; seven participants (43.8%) reported non-suicidal self-harm in the past week and six (37.5%) participants reported non-suicidal self-harm in the past year; seven participants (43.8%) reported suicidal ideation in the past week and five participants (31.3%) reported suicidal ideation in the past year.

### Main findings

Four TFA domains emerged as explanatory factors of determining the acceptability of the volitional help sheet for self-harm: affective attitude, burden, intervention coherence and perceived effectiveness. The domains ethicality, opportunity costs and self-efficacy were not discussed in sufficient detail by participants, and were therefore deemed not be indicators of acceptability. Explanatory themes are provided with respect to each domain, with accompanying illustrative quotes.

### Affective attitude

Participants described positive attitudes with respect to using the volitional help sheet for self-harm. Participants believed that using the volitional help sheet would help people to identify both problematic situations when people may have self-harmed in the past, and specific responses that might help to support people in reducing self-harm. Participants also described the positive aspects of being able to interact with the tool, to form coping plans as a way of proactively managing their response to specific situations where they may feel the urge to self-harm.

‘Yeah, I found it really helpful, because it's not something that I'd particularly considered before. I've had it mentioned to me by other professionals in the past, but it's kind of the information that sort of bounces straight off. So, having it kind of laid out in front of me, was really helpful.’ p21

Participants described how they found the volitional help sheet particularly helpful for reminding them about the techniques they could try to help them avoid self-harming. Having multiple coping plans to try when they feel the urge to self-harm was also perceived as an advantage of the tool, rather than having one particular technique that may not always work. Participants also described how the tool could be used as a prompt or a reminder to implement coping plans that could be used, but could also raise their own awareness of things they may already be doing (as coping plans), without explicitly realising they are doing so. Consequently this was perceived as helping them to formalise these plans.

‘It did remind me that there are some quite simple things I can do like speaking to someone I trust because I do that on a regular basis, but it does remind you that you can do something simple.’ p03

Participants identified a number of considerations and challenges for wide-scale use of the volitional help sheet. Participants described how the volitional help sheet would be helpful for people in certain situations and certain contexts, such as being in the right ‘frame of mind’ to engage with the tool. Further, participants emphasised that readability should be an important consideration, to ensure that participants have a sufficient understanding of the content and purpose of the intervention. This was especially true in cases where the intervention might be delivered by a healthcare professional, where the purpose of the intervention could be clearly communicated to patients as part of efforts to engage participants in the intervention.

‘I've never seen them before, so they were completely new to me. I was, sort of, looking at them and I was thinking, if it was someone who was in quite a poor state of mind, they may not actually understand the purpose of them. It, kind of … I mean, I have dyslexia … but it all, sort of, went, whoosh, for the purpose behind them and what any professional would, sort of, get from that.’ p20

However, participants had different views about whether the tool could be used in ‘emergency situations’, such as when a person has recently self-harmed.

‘I tell you what, I wouldn't give this thing to somebody who literally has just, like, attempted suicide, like, in the last couple of months, do you know what I mean? Because that would be, it's a bit … you've got to have a certain amount of self-awareness, do you know what I mean? To actually carry out those steps, if it was too raw, it's not going to happen.’ p18

One participant described how the tool could be particularly important for people who have other long-term health conditions, which may present additional challenges and consequently affect how people engage with the tool, such as using specific responses to certain situations. More generally, having a tool that was easy to engage with was described as an important factor in people's motivation to use the tool for its intended purpose.

‘Yes, I find them helpful at times. It depends on like the situation or the context of what's going off around me. So I don't know if you're aware but anorexia can be sometimes considered as an emotional coping mechanism so is self-harm which can be quite prolonged in usage and things like that and can leave quite a lot of damage so, yes.’ p08

Although most participants believed the volitional help sheet was straightforward and easy to use, a potential challenge was that not all situations and solutions would be perceived as relevant to everyone. Participants reported that this might act as a distraction and consequently impede the perceived helpfulness of the volitional help sheet. Therefore, the challenge is to ensure that the instructions could sufficiently engage people.

‘I mean, it was pretty straightforward, I understood it, some of the, if questions, like, if you self-harm to get attention then, some of them were, I remember finding them a little bit presumptuous, like, it's not the reason. And I would have liked there to be an option, like, reason doesn't apply to me or something like that.’ p18

### Burden

Participants described how the volitional help sheet may be perceived as burdensome when in particular situations where they might find it difficult to concentrate and engage with the tool. This could be during particular situations where negative feelings may be heightened. Participants reported that the volitional help sheet would take less effort for people who were in the state of mind where they were motivated to try and implement strategies aiming to avoid self-harming.

‘Yeah, and also I think it's difficult, because depending on your frame of mind at the time, if I'm feeling a bit like I'm just not managing very well at that time, I mean, right now I'm managing probably at my best, I guess, but in a period where I'm not, then I could very easily feel quite crappy about myself and thinking that I don't do those things, maybe, or – I don't know – it would just be … and also it would just be a job to get done, but I wouldn't get done. And I would feel about not getting done.’ p11

Participants described how they could incorporate their coping plans into their daily routine. This was particularly true in raising awareness of coping strategies and having something that was easily available (such as reminders about contacts for local support services) that could be used for their personal circumstances.

‘If-then plan is great along with a lot of the other grounding techniques but when you're feeling really stressed and about to self-harm, you're not thinking that so you probably need something that's going to go, ah, yes.’ p10

Participants identified that being able to establish automatic coping plans would be beneficial with respect to incorporating them into their daily routine and increasing the frequency with which they are used. The importance of being able to establish coping plans as habit was seen as a crucial factor in ensuring coping plans are used and referred to over the long-term.

‘And over time, you might start changing the patterns of your thoughts to do so, and especially if you look at it fairly regularly. Trying doing the long term, hopefully, yeah, it would help. I don't know what word I want to use, but alter your behaviour.’ p12

### Intervention coherence

Generally, participants reported that they understood the volitional help sheet and how it works. The statements themselves and the accompanying instructions were perceived as being clear and understandable. Some participants described some of their past experiences making ‘safety plans’, which they compared to forming implementation intentions, and were therefore familiar with the process involved.

‘Yeah, no, it's helpful, so I've done, kind of, safety plans and things and so at certain points I've got a lot of insight into it, how I am, so I can, you know, my safety plan was, kind of, if I'm preventing my if's then I need to do this, and then … so similar, so at different, depending on how I'm presenting, what bit I would need to do, what my thinking is, I guess, at that point.’ p04

One participant reported how the volitional help sheet empowered them to be more proactive, and helped them develop their own strategies to try and reduce self-harm.

‘So, it just seemed very straightforward and it really gave me new ideas about how I could address those behaviours. And I also felt that the, kind of, it was very practical and felt quite, kind of, empowering, so I just thought it was a really useful resource and, for me, it seemed like something I was, kind of, really glad I'd come across it and actually has stuck with me since.’ p16

Some participants reported that some of the situations may not be applicable or familiar to everyone, which may affect how people understand the volitional help sheet and its intended purpose. More generally, not being familiar with the overall purpose of the volitional help sheet may affect how people respond to them, therefore emphasising the importance of clear instructions. Consequently, participants suggested improvements to the intervention that may further increase its acceptability with respect to how well people understand the purpose of the intervention. Some participants highlighted reminders about signposting to support services as an important feature of the volitional help sheet. To further strengthen this aspect, including signposting to other support services was perceived as potentially helpful, and an option that could be further developed or amended.

‘And I think also perhaps having the ability to … what am I trying to say … to adapt or modify these things to yourself, because it might not be the Samaritans that would be their go to, it might be something else, for example.’ p11

Overall, participants were positive about most of the included statements within the volitional help sheet. However, the option to amend the existing statements to make them more personally relevant was perceived as a potentially useful feature.

‘Yes. I mean, maybe if there was a feature for rewording them so that they were in your own language you might take them in a bit more, I don't know.’ p03

The option to rank their own implementation intentions was seen as a helpful additional feature. Participants believed that this might be a way of being able to identify which statements were the most applicable to them, and also to provide several coping plans to try if earlier ones were not successful.

‘You ask yourself what things are more likely to help than not, so ranking them might be helpful, instead of being, like, here they all are, pick one.’ p18

Offering more flexibility in the construction of if-then plans was perceived as particularly important for participants who did not engage with the intervention. One participant suggested that providing people with the opportunity to add or amend existing statements might be one way of increasing engagement.

‘So, personally, that is, I think, how I would prefer to see it rather than as a fixed template that's being presented as a very open … you know, here are some suggestions, this is what we found helpful in the past maybe with other people, now let's go and build yours.’ p10

Emphasising in the instructions that not all situations and solutions may be applicable to everyone was suggested as a helpful addition. Further, involving examples or case studies of how people have used them in their daily routines was also seen as a useful addition.

‘Yeah, and more relevant, yeah, and useful, yeah. I think also one thing I was thinking about is that if you had like perhaps incorporated in some way examples of how people had actually applied it in their life, the situation bit, and how, you know, how they'd … that they'd found it useful, that would make it feel more encouraging and human.’ p11

### Perceived effectiveness

Participants recognised the opportunity for the volitional help sheet to be used by healthcare professionals as part of healthcare delivery for people with a history of self-harm. Different healthcare professionals were discussed, and mental health professionals were highlighted as a particular group who might benefit from using the volitional help sheet with patients. The therapeutic interaction was perceived as an enabler of being able to use the volitional help sheet as part of therapy. Participants explained how the volitional help sheet could be used to supplement the healthcare that patients are already receiving.

‘I think … so in therapy I think it's useful to sort of go through the different strategies that you can use, so like safety planning is one of the things that gets brought up a lot, or distraction techniques and harm minimisation or whether I've got thoughts on those, so having it sort of … I suppose if you're doing therapy, having it as something that you can take away with you as a reminder, I wonder how useful it might be as like an app form.’ p13

Using the volitional help sheet as part of existing healthcare was suggested as a potential way to increase uptake of the intervention among people who did not engage with the intervention.

‘If they were unable to communicate to the keyworker or to a healthcare professional, they could give that diary so then they were able to monitor and see where the triggers lie and stuff and then help to explain to them where that might be coming from.’ p08

The volitional help sheet was also perceived to be useful as a tool or resource that could be used to prompt a discussion about strategies to reduce self-harm. Participants suggested this could be made available in general practitioner (GP) surgeries, for example, or used directly by GPs during patient consultations; participants noted that the brief nature of the intervention meant it could be feasible to use during a time-restricted GP consultation.

‘I also thought, maybe in, like, GP surgeries in waiting rooms, might be the kind of thing people pick up.’ p16

Many participants believed intervention effectiveness was likely to be dependent on people's willingness to engage with the intervention, and being in the ‘state of mind’ to engage with the content of the volitional help sheet.

‘So, I always think the best time to create something like this, is when you're in quite a good state of mind, where you have quite good insight.’ p21

Being able to recognise when people may need support to reduce self-harm was perceived as important. Further, the importance of being able to establish new habits was reported as being an important part of perceived effectiveness.

‘If-then plans I would say only work for people that have accepted they have an issue to deal with and it takes someone that has the knowledge that they need to deal with something, it's not applicable to them.’ p14

Participants described how the volitional help sheet was particularly effective for raising awareness of where and when the urge to self-harm may be heightened, and to identify appropriate coping strategies.

‘Because then I think people who do suffer with it quite regularly would be able to track and then say if they were unable to communicate to the keyworker or to a healthcare professional, they could give that diary so then they were able to monitor and see where the triggers lie and stuff and then help to explain to them where that might be coming from, yes, things like that.’ p08

Participants also believed it would be helpful in establishing automatic coping plans over the long term. This was also helpful as a reminder strategy for people to refer to their coping plans when the urge to self-harm may be heightened, or at perceived times of crisis.

‘Yeah, I think it could help in the long term. Because it gives you new ideas, it could be a real source of help, I guess it leaves you, kind of, in control of how you want to address that problem.’ p16

Being able to construct their own personalised coping plans was reported as a key strength of the volitional help sheet, and was perceived to be key to its effectiveness.

‘The thing I liked about the if-then plans is, like, the ownership you have over them, whereas everything else, like my care plan's written for me, even though I have input.’ p04

## Discussion

This study aimed to apply the TFA to (a) explore peoples’ experiences of a brief online theory-based intervention to prevent and reduce self-harm; and (b) understand the most prominent aspects of intervention acceptability, to make recommendations for intervention refinements and ensure successful implementation of the intervention. To our knowledge, this is the first time the TFA has been applied to understanding acceptability of interventions for self-harm. This study makes two important contributions to the literature. First, we describe four prominent TFA domains (affective attitude, burden, intervention coherence and perceived effectiveness) derived from a qualitative analysis of people who completed an online questionnaire containing an intervention designed to help prevent repeat self-harm, which provide insight into determining the acceptability of the volitional help sheet for self-harm and may further increase intervention effectiveness. Second, the study describes refinements that could be made to further increase intervention acceptability, and we present subsequent recommendations as to how the intervention could be rolled out more widely to people with a history of self-harm.

Although people were positive about using the volitional help sheet, we found some inconsistencies in attitudes about whether the volitional help sheet could be used in ‘emergency situations’, such as when a person has recently self-harmed (affective attitude). This is surprising, given that the volitional help sheet for self-harm has previously shown some level of effectiveness among people recently admitted to hospital.^6,29^ However, this suggests a need to consider both effectiveness and acceptability when designing interventions, to ensure sufficient uptake by those people who would benefit. Further, our previous study examining associations between demographic variables and acceptability domains according to the TFA found no differences in acceptability of the intervention based on recent history of self-harm.^19^ One possible explanation may be that our findings suggest that people believe that not all of the situations and solutions may be applicable to everyone. Consequently, further refinements and acceptability testing may be required specifically among people with a more recent history of self-harm. This would also ensure that our intervention considers the variation in history of self-harm.

Engagement was highlighted as a motivating factor in using the volitional help sheet (perceived burden). Our participants described how some people who have recently self-harmed may not always be in the right ‘state of mind’, which may affect engagement. Future research could therefore examine whether making the intervention available ‘offline’, for example, may increase acceptability. Interventions for mental health problems delivered online are considered to be highly acceptable,^30^ and with further refinements, our intervention could be developed to include an offline feature to ensure people can engage with the intervention at a time convenient for them.

Participants in our study generally understood the volitional help sheet and how it worked (intervention coherence). Participants outlined additional features that would be helpful, including signposting to support services for self-harm, and the option to amend the existing statements to make them more personally relevant. Signposting to support services and the ability to personalise content have previously been shown to be an acceptable component of technology-based interventions for suicide prevention,^31^ and thus could be explored in future iterations of the volitional help sheet.

Participants raised the issue of implementation of the volitional help sheet by healthcare professionals (perceived effectiveness). The healthcare professional–patient interaction was reported as a potential enabler of using the volitional help sheet as part of interactions with a healthcare professional. This is consistent with wider research emphasising the importance of the healthcare professional–patient relationship in providing the platform for professionals to talk to patients about their health.^32^

### Implications for practice

Our findings suggest that the volitional help sheet for self-harm was perceived as acceptable among people who have previously self-harmed. Further developments and recommendations for implementation would be helpful in four areas. First, further exploration is needed to further increase acceptability among people who have more recently self-harmed. Second, wider-scale roll out must include ways of making the intervention more accessible offline, and therefore not requiring internet access. Third, additional features of the intervention should be considered, such as links to support services. Fourth, further examination is needed into how the intervention could be delivered by healthcare professionals to support people in reducing repeat self-harm.

Research suggests more emphasis should be placed on improving care for patients who have harmed themselves, with a focus on improving the implementation of clinical guidelines.^33^ It is encouraging that participants identified the potential role that healthcare professionals could play in helping to support people in reducing self-harm, given the importance of healthcare services in self-harm and suicide prevention strategies. Implementation intentions offer a brief theory-based intervention that could be feasibly incorporated into time-restricted medical consultations and safety plans. Important considerations for implementation include ensuring the purpose of the intervention is clearly communicated to patients, and ensuring the responses are protective rather than triggering for patients. Future research could explore the extent to which these brief interventions could be delivered as part of GP care, given the recognition of primary care as being an important place to help people in reducing repeat self-harm.^34^

### Strengths and limitations

In using the TFA,^8^ our study provides a robust theoretical basis for future studies aimed at further developing the volitional help sheet, both in the context of self-harm and other health contexts. Four TFA domains emerged as indicators of acceptability of the volitional help sheet for self-harm: affective attitude, burden, intervention coherence and perceived *effectiveness*. Using the TFA instead of more general approaches to exploring acceptability allows for a more rich and varied assessment of how people think and feel about taking part in interventions.

There are limitations to this study. Participants in the present study had previously taken part in a cross-sectional survey of people who had previously self-harmed, and had volunteered to be interviewed. Consequently, the sample may not be representative of all people who have previously self-harmed and there may be additional views that were not captured in the present sample. Further, our sample also contained participants who did not engage with the intervention (*n* = 3). Although not the focus of the present research, it may be valuable for future research to further explore reasons for non-engagement. Additionally, our sample was predominantly White British. Although we were able to identify potentially important indicators of intervention acceptability, we were unable to compare views and opinions across ethnic groups, which could be examined in future research.

In conclusion, a brief intervention based on implementation intentions has been shown to be effective in reducing self-harm in people recently admitted to hospital after an episode of self-harm.^6^ Our findings suggest perceptions of acceptability more generally among a community sample of people who have previously self-harmed, explained by four TFA domains: affective attitude, burden, intervention coherence and perceived effectiveness. Further modifications could still be made, but it is hoped that this intervention provides a useful tool for both individuals to construct their own personalised implementation intentions, and as part of longer-term support for preventing self-harm as delivered by healthcare professionals.

## Data Availability

The data that support the findings of this study are available from the corresponding author, C.K., upon reasonable request.

## Supplementary material

For supplementary material accompanying this paper visit http://doi.org/10.1192/bjo.2022.568. click here to view supplementary material
