# Supplementary material for: Exploring the acceptability of a brief online theory-based intervention to prevent and reduce self-harm: a theoretically framed qualitative study
Source: BJPsych Open. 2022 Oct 12;8(6):e184. doi: 10.1192/bjo.2022.568 (PMC9634605; doi:10.1192/bjo.2022.568)
Supplement: Supplementary file 1 [file S2056472422005683sup001.docx]

***Using “IF-Then plans” to help people avoid self-harming: Qualitative study to assess acceptability of a psychological intervention***

**Interview topic guide***

| **Attitudes towards making IF-THEN plans** |
| --- |
| - How did you feel about taking part in the intervention?   - What were the positive aspects of making IF-THEN plans to help you reduce self-harm?   - Were there any challenges with making IF-THEN plans?   - Is action planning something you find helpful? Have you tried action planning previously? |
| **Burden of making IF-THEN plans** |
| - How much effort was required to make your IF-THEN plans?   - How were they incorporated into your daily routine, if at all?     - Could you provide an example of how you did this? |
| **Ethicality of making IF-THEN plans** |
| - How much was forming IF-THEN plans a good fit with your personal values? How do you feel about making IF-THEN plans generally?   - Probe good fit versus challenging to align them with personal values |
| **Intervention coherence** |
| - To what extent do you understand IF-THEN plans and how they work?   - What aspects were clear? Were there any parts that were unclear? Could you suggest any improvements to the intervention? |
| **Opportunity costs** |
| - To what extent did you give up any benefits, profits or values to make your IF-THEN plans, and to engage in these plans? |
| **Perceived effectiveness** |
| - How confident were you / are you that your IF-THEN plans are likely to support you and other people in helping to reduce repeat self-harm? |
| **Self-efficacy** |
| - How confident were you about making your IF-THEN plans to support you in reducing repeat self-harm? - How confident are you about maintaining your IF-THEN plans? |

*Topic guide will be developed/modified iteratively depending on participants’ responses to the questionnaire
